# Rereading the genetic origin of cancer: the puzzle of all eras

**DOI:** 10.2144/fsoa-2022-0014

**Published:** 2022-05-12

**Authors:** Berjas Abumsimir, Talal S Al-Qaisi, Yassine Kasmi

**Affiliations:** 1 Department of Medical Laboratory Sciences, Pharmacological & Diagnostic Research Centre (PDRC), Faculty of Allied Medical Sciences, Al-Ahliyya Amman University (AAU), Amman, 19328, Jordan; 2 Johann Heinrich von Thünen Institute, Bundesallee, 50/38116, Braunschweig, Germany

**Keywords:** cancer, evolution, multicellular organism, mutation, risky alleles

This editorial sets out to scientifically review the concept of cancer from a purely genetic perspective in which we try to link the development of tumors to the driving force of the evolution of living beings: mutations. The aim is to introduce the point of view that cancer diversity has parallels to the whole developmental process of the cell, attempting to expand the rolling concept of cancer. This may contribute to altering our view of this dangerous biological phenomenon that has arisen on the margins of the evolutionary process.

When Charles Darwin (1809–1882) and Alfred Wallace (1823–1913) announced their research about natural selection, opponents asked for evidence supporting this driving force for adaptation in response to natural environments. The supporters replied that mutations can change the organism’s traits; that is, there are positive mutations. In contrast, ‘pathogenic variants’ should also exist. The random manner of environmental factors that affect organisms does not take into account the positives or negatives; instead, it just works alongside the complex response mechanisms that produce mutations on the gene level. These mutations might block the gene’s activity or may improve the production of the gene. Regardless of the reasons for their genesis, new mutations always appear as evolution continues, and the number of mutations increases continuously. Hypothetically, early *Homo sapiens* had an unknown number of mutations, and over time the number of mutations is distributed vertically by increasing the number of mutations in every generation, and horizontally by increasing the number of individuals carrying it in a population. Every new mutation will not be found in the ancestor, but it will appear in the next generations/populations. Logically, if the genetic risk of a disease or disorder is distributed widely among human populations, the mutation or mutations that cause the disease should have an ancient origin and *vice versa*: if a genetic defect is excluded from a particular population, that means the mutation has a modern origin.

An unknown number of ‘pathogenic variants’ will cause diseases such as tumors. In the context of the big picture of evolution, cancer seems to be one of the side effects of the evolutionary process on the genetic level. Many diseases and genetic disorders including cancer are very old, and new types of cancer will continue emerging. This explains why certain diseases are endemic in certain populations and why cancers affect all human populations on the earth.

The story of cancer continues across biological ages; some studies have confirmed the existence of cancer in other mammals [1,2]. Could we then conclude that we have inherited risky alleles from our mammalian ancestors? If the answer is yes, then we should declare that the mutations causing lethal tumors may be in one or more of the following categories: mutations exclusive to certain populations (modern origin); mutations common in all humans (old origin); some mutations common in all primates (very old origin); some mutations common in all mammals including primates (ancient origin); or (E) mutations that developed during the evolution of multicellular organisms (very ancient origin).

Heredity as a biological process includes everything that can be inherited, not only risky alleles and the inheritance of the genome as a whole, so adding the concept of ‘risky evolutionary support’ through biological time seems very appropriate here. This means that the increasing complexity of the organism leads to the increase of spontaneous errors in the DNA. Furthermore, the increasing complexity of the organism means an increase in the complexity of major biological processes such as growth, movement and response, consequently leading to increased exposure to internal and external factors that are very likely to increase nucleic acid errors, and this is directly reflected not only in the emergence of tumors but also in their diversity.

Are tumor mutations exhibiting a parallel route with evolution (complexity of cell regulation, increasing of organ functions, increasing numbers of proteins produced etc.)? If so, from a direct perspective, the tumor will be more diverse over time.

For instance, breast cancer is one of the most common types of tumors among females in the world. One of the genetic risk factors is the status of the *BRCA1* gene, where some imbalances are found to be increased in patients with the tumor; certain inherited mutations in this gene increase the chance of developing the tumor [3,4]. Similarly, some men with prostate cancer have been found to have specific *SPOP* mutations; the mutations in *SPOP* are not limited to specific human origins and are not limited to a geographical area [5,6]. These are examples of inherited mutations that involve the majority of the human race, which means that the inheritance of these mutations is old. However, there are exceptions here that require reflection. For example, the *HOXB13* gene, specifically the G84E germline mutation, has been found to be associated with an increased risk of prostate cancer among individuals in some European ancestries [7,8]. In contrast, this point mutation does not constitute a serious risk for the Chinese [9] or Jewish populations [10], although there are other mutations in the same gene that may lead to prostate cancer in the above-mentioned populations.

It seems that cancer must be a phenomenon that pre-dates the evolution of animals, if we look at it from a purely genetic point of view. Mutations that cause tumors in the first place are only a genetic manifestation of evolution, a transient process of genus and species. To understand the mechanisms of tumor development, we must analyze the genetic origin of tumors.

The complex processes of evolution impose on living cells certain kinds of changes that are unavoidable to keep pace with the complexity of these factors, and this imposes a greater complexity in the processes of cellular regulation and production of proteins because the responses to these factors differ from one individual to another within living societies. If these events are old, resulting in inherited genetic changes that were appropriate to prevailing conditions at the time, these genetic mutations may not be suitable for emergency conditions or are inefficient in the face of new genetic challenges.

Additionally, genetic errors occur at the organizational level. These genetic errors cause tumors with the highest degree of severity, depending on where the mutations occur within the genome. Given that the different changes at the genetic level are a way to change the genome’s structure toward adapting to cell-emergent conditions that ultimately lead to evolution, the question remains to be answered: are tumors a partial mechanism of evolution, or are they only side effects of the whole process?

We find that for those who have a particular cancer and have a family history of the disease, the rate of emergence of certain mutations – especially the most dangerous ones – is greater than in those who do not have a family history of that disease [11,12]. These gene mutations may be nonsense or frameshift mutations that lead to the emergence of an inactive partial protein, missense mutations that replace one amino acid with another, having an unknown effect on the protein’s biochemical properties, or synonymous silent mutations with limited impact. For instance, in one of the common tumors, prostate cancer, the serious K1019X (3055A>T) nonsense mutation of the *EPHB2* gene was found to be present in 15.3% of the African Americans with positive family history, compared with a lower percentage among healthy African American male controls (5.2%) [13]. There are other aspects, such as that some populations have their own dangerous inherited alleles instead of different types of tumors, but the examples are more than we can mention here.

The proportion of inherited tumors is large and cannot be counted if we try to collect evidence on the genetic origins of cancer and redefine it in relation to this phenomenon, which is almost purely genetic. This definition may mean exploiting the genetic basis and finding new ways to think about cancer. The goal of changing perspectives is to reduce the incidence of this deadly disease. The main dilemma is to provide a suitable definition of cancer regarding its genetic origins, which leads directly to the correct verification of its causes and this opens the doors to deal with it properly.

The modern definitions of cancer agree that it is a group of diseases that lead to a defect in cellular growth, with the possibility of spreading this imbalance to neighboring cells. Hippocrates (460–370 BC) was the first to call these tumors ‘cancer’ because of their anatomical resemblance to a crab, and because Hippocrates believed that bodily fluids consist of various types of ‘Chemos’, including black bile and yellow bile, those who came after him took a similar approach to explain the causes of cancer. The definitions of Galen (129–210 AD) and Avicenna (980–1037 AD) suggest that cancer happens as a result of increased ‘black blood’ that settles in a body organ to form the tumor and spreads through the tumor’s veins from the center of the tumor to neighboring organs.

Modern medicine also recognizes the importance of circulating tumor cells for certain tumors. But the difference between old knowledge and modern knowledge of cancer is the tremendous discoveries that have been made in genetics and molecular biology, and the ability to predict genes’ actions at the cellular regulatory level.

The definition given above has lasted for centuries. Interestingly, it focuses on the metastatic process of the tumor (in modern terminology); the old scientists were well aware that something was causing the size of the tumor to increase, which led to the same modern definition that focuses on a defect in cellular growth with the possibility of metastasis in the body. A new revision of the definition of cancer needs to take into account the biological basis of the evolution associated with genetic changes and trace the genetic origins of tumors. It is time to take a bold step into new horizons regarding the concept of cancer and its association with biological evolution and to study intensively whether tumors are a response to the complex objective conditions imposed by evolution in the form of inherited genetic mutations.

In light of what we have mentioned above, is it also possible to consider that tumors are the final form of development at the cellular level? Cancer can be defined as an inevitable price of the everlasting development of living beings, which is not limited to humans but originated with the emergence of multicellular organisms. We recommend here that further discussions are necessary on the way in which tumors are intertwined with evolution and the emergence of different mutations in the direction of establishing a new concept amid the human struggle against cancer, the puzzle of all eras.
